# Enriched environment ameliorates postsurgery sleep deprivation‐induced cognitive impairments through the AMPA receptor GluA1 subunit

**DOI:** 10.1002/brb3.2992

**Published:** 2023-04-24

**Authors:** Jie Gao, Lina Zhao, Dedong Li, Yun Li, Haiyun Wang

**Affiliations:** ^1^ Department of Anesthesiology the Third Central Clinical College of Tianjin Medical University, Nankai University Affinity the Third Central Hospital, Tianjin Key Laboratory of Extracorporeal Life Support for Critical Diseases, Artificial Cell Engineering Technology Research Center, Tianjin Institute of Hepatobiliary Disease Tianjin China; ^2^ Department of Anesthesiology Tianjin Haihe Hospital Tianjin China

**Keywords:** BDNF, enriched environment, glua1, postsurgery sleep deprivation

## Abstract

**Background:**

As a common postsurgery complication, sleep deprivation (SD) can severely deteriorate the cognitive function of patients. Enriched environment (EE) exposure can increase children's cognitive ability, and whether EE exposure could be utilized to alleviate postsurgery SD‐induced cognitive impairments is investigated in this study.

**Methods:**

Open inguinal hernia repair surgery without skin/muscle retraction was performed on Sprague‐Dawley male rats (9‐week‐old), which were further exposed to EE or standard environment (SE). Elevated plus maze (EPM), novel object recognition (NOR), object location memory (OLM), and Morris Water Maze assays were utilized to monitor cognitive functions. Cresyl violet acetate staining in the Cornusammonis 3 (CA3) region of rat hippocampus was used to detect neuron loss. The relative expression of brain‐derived neurotrophic factor (BDNF) and synaptic glutamate receptor 1 (GluA1) subunits in the hippocampus were detected with quantitative reverse transcription polymerase chain reaction (RT‐qPCR), Western blots, enzyme‐linked immunosorbent assay (ELISA), and immunofluorescence.

**Results:**

EE restored normal levels of time spent in the center, time in distal open arms, open/total arms ratio, and total distance traveled in the EPM test; EE restored normal levels of recognition index in the NOR and OLM test; EE restored normal levels of time in the target quadrant, escape latencies, and platform site crossings in the Morris Water Maze test. EE exposure decreased neuron loss in the CA3 region of the hippocampus with increased BDNF and phosphorylated (p)‐GluA1 (ser845) expression.

**Conclusion:**

EE ameliorates postsurgery SD‐induced cognitive impairments, which may be mediated by the axis of BDNF/GluA1. EE exposure could be considered as an aid in promoting cognitive function in postsurgery SD.

## Graphical abstract

1. EE ameliorates post‐surgery SD‐induced cognitive impairments, which may be mediated by the axis of BDNF/GluA1. 2. EE exposure could be considered as an aid in promoting cognitive function in post‐surgery SD.

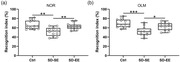

## 1 INTRODUCTION

Sleep deprivation (SD) can result in decreased cognitive and memory function, inadequate alertness, and health deterioration, defined as insufficient sleep relative to one's usual baseline (Abrams, 2015; Cavallo & Mallory, 2004; Dolan et al., 2016). As an ordinary aspect of society, the prevalence of SD is increasing. Regular SD increases the risk of diabetes, heart disease, depression, and enhanced impulsive behaviors by causing hormonal imbalances (Banfi et al., 2019). Some studies have testified that SD impairs the consolidation of motor‐adaptive working memory, declarative memory, and hippocampal‐dependent emotional memory (Iranzo, 2022; Siegel, 2022). Mechanically, SD may inhibit the synthesis of long‐term potentiation stabilization‐related protein from inducing long‐term potentiation (Sur & Lee, 2022).

SD often occurs in patients after surgery, especially major surgery, which demonstrates a potentially deleterious effect on postoperative recovery. Age, comorbidity, the severity of surgical trauma, type of anesthesia, and postoperative factors are the main factors associated with postoperative SD (Gao et al., 2022; Su & Wang, 2018). Sleep hygiene interventions, such as sedation, environmental noise reduction, daytime activities promotion, and enriched environment (EE) exposure, can alleviate SD (Li et al., 2019).

EE offers many opportunities to stimulate the brain through the physical and social surroundings compared with the standard environment (SE) (Sale et al., 2009). EE has long been lauded as a strategy to increase children's cognitive ability, learning and memory, and well‐being in educational contexts. On the other hand, EE can also reduce the reactivity to stress and anxiety (Ball et al., 2019). Mechanically, EE induces neural and synaptic plasticity to promote neural transmission, neural density, and dendritic branching (Gao et al., 2020). In our previous research, we testify that EE ameliorates spatial memory impairments after postsurgery SD in rats, which demonstrates the utilization of EE to alleviate surgery SD‐induced memory impairments (Gao et al., 2020).

As the most common excitatory glutamate receptors coupled to calcium and sodium ion channels, α‐amino‐3‐hydroxy‐5‐methyl‐4‐isoxazolepropionic acid receptor (AMPAr) mediate signals transduction between the neuronal circuits of the hippocampus and promote fast excitatory synaptic transmission. It is now identified that AMPAr involves glutamate receptor (GluA) 1–4 (Chen et al., 2022). Among these, GluA1 is a crucial mediator of hippocampal synaptic plasticity. On the other hand, the early onset of cognitive impairment in mice depression model is associated with enhanced hippocampal GluA1 expression and altered synaptic plasticity (Gross et al., 2015). The potential role of GluA1 in postsurgery SD‐induced cognitive impairments is not deciphered.

Our analysis demonstrates that EE exposure can improve postsurgery SD‐induced cognitive impairments with upregulated brain‐derived neurotrophic factor (BDNF) and GluA1 expression, which indicates that EE exposure could promote postsurgery SD‐induced cognitive impairments through BDNF/GluA1 pathway.

## 2 METHODS & MATERIALS

### 2.1 Rats

Sprague‐Dawley male rats (9‐week‐old, 240−260 g) were classified into three groups (*n* = 12): Ctrl group (rats housed in SE without treatment), SD‐SE (after hernia repair surgery, rats housed in SE with a 2‐day SD exposure), and SD‐EE group (after hernia repair surgery, rats housed in EE with a 2‐day SD exposure). All the animal experiments were approved by the Institutional Animal Care and Use Committee of Tianjin Medical University. Three batches of rats were utilized to verify the consistency of the results.

### 2.2 Postsurgery sleep deprivation

Open inguinal hernia repair surgery without skin/muscle retraction model was constructed as described in our previous report (Gao et al., 2020). Briefly, rats were intra‐peritoneally anesthetized with xylazine (0.005 mg/g) and ketamine (0.1 mg/g), and the medial thigh on the left side was shaved. The skin, approximately 4 mm medial to the saphenous vein, was cut into an incision (15−20 mm). A 7−10 mm long incision was further cut in the superficial muscle layer, underneath which a retractor (Biomedical Research Instruments Inc., USA) was inserted to position all prongs. Then, the site of hernia repair surgery was closed with 4.0 Vicryl sutures. Tactile stimulation was achieved with a horizontal bar sweeping just above the cage floor from one side to the other side of the mouse cage. A sleep fragmentation chamber was used to induce severe SD; the interruption mediated by the sweeper was set as constant and lasted for 48 h (intersweep cycle values, 15 s).

### 2.3 Environment exposure

According to previous reports, SE and EE exposure were set up (Crawford et al., 2020; Requejo et al., 2018). SE rats were housed in standard rat wire‐topped clear plastic cages (52 × 36 × 20 cm), with full access to food and water; EE rats were cultured in larger rat plastic cages (70 × 45 × 38 cm), in addition to food and water, various objects, such as running wheels, stairs, a tunnel, a small compartment, and different shaped and sized objects with different sizes were provided. Objects in the EE were changed daily and placed at different spatial locations of the cage.

### 2.4 Elevated plus maze

The elevated plus maze (EPM, Noldus, Beijing, China) apparatus was placed about 60 cm above the ground, which consisted of two closed arms (36 cm × 6 cm) and two open arms (36 cm× 6 cm) emanating from a common central platform (6 cm × 6 cm). Rats were positioned at the intersection of the closed and open arms, facing the closed arms. The exploratory behavioral test was performed in the dark phase in combination with EthoVision XT video tracking for 5 min. The times spent in the center, time spent in the distal open arms, open/total arms ratio, and total distance traveled were measured.

### 2.5 Novel object recognition and object location memory assay

The novel object recognition (NOR) task (Creative Biolabs, Hongkong, China) was utilized for the assessment of memory alterations. During the training phase, two identical objects were placed on the opposite side of the task. The rats were then placed in the center of the task, adapting for 4 min. Then, 24 h later, rats were put back in the same apparatus and exposed to a familiar object A and a novel object B. The behavior of rats was recorded for off‐line analysis by an experimenter blind to the treatments. The recognition index was calculated as the proportion of time with the B object out of the total time of A and B. Twenty‐four later, the object location memory (OLM) task was performed in the open field arena utilized in the NOR test, and rats were allowed to explore the arena for 5 min. Then the positions of A object and B object were switched. Twenty‐four later, the behavior of rats was further recorded.

### 2.6 Cresyl violet staining

Cresyl violet staining was utilized to detect Nissl substance in the cytoplasm of the neuron. Briefly, the paraffin‐embedded Cornusammonis 3 (CA3) region of rat hippocampus was coronally sectioned into 2 μm slides, which were stained with 0.1% cresyl violet acetate (Scientific Phygene, Fuzhou, China). Well‐rounded neurons with visible nucleoli were observed with an Olympus AX70 microscope and counted with the NIH‐Image J1.51p 22 software.

### 2.7 Real‐time polymerase chain reaction

Hippocampus tissues were harvested and homogenized with TRIzol Reagent (Invitrogen, Waltham, MA) to obtain the total RNA, which was reverse‐transcribed with a RETROscript Reverse Transcription Kit (Invitrogen). Real‐time polymerase chain reaction (RT‐PCR) was performed with SYBR Green PCR Master Mix (Thermo Fisher Scientific, Waltham, MA). The reaction procedure was set: 95°C for 10 min, 40 cycles of 95°C for 15 s, and 60°C for 1 min. Expression data were normalized to *Gapdh* and calculated with the ΔCt method. The following primers were used: *Gapdh*, 5′‐TGTAGACCATGTAGTTGAGGTCA‐3′ (forward) and 5′‐AGGTCGGTGTGAACGGATTTG‐3′(reverse); *Bdnf*, 5′‐GCCTTCATGCAACCGAAGT‐3′(forward) and 5′‐GAGGGCTCCTGCTTCTCAA‐3′ (reverse).

### 2.8 Synaptosome preparation and Western blot

For synaptosome preparations, hippocampus tissues were gently homogenized in ice‐cold sucrose buffer (0.32 M, pH 7.4). The relevant supernatant was continuously centrifuged at 1000 × *g* (10 min, 4°C) and at 10,000 × *g* (20 min, 4°C) to obtain synaptosome. Then, the synaptosomal pellet was resuspended with lysis buffer (0.1% Triton X‐100, 25 mM KCl, 150 mM NaCl, and 10 mM Tris‐HCl [pH 7.4] with PMSF) at 4°C for 10 min.

The synaptosome was separated by 10% sodium dodecyl sulfate–polyacrylamide gel electrophoresis and transferred to polyvinylidene fluoride (PVDF) membranes. After nonspecific blocking with 5% nonfat dry milk, the PVDF membranes were incubated with the primary antibodies against BDNF, β‐actin, synaptosomal (Syn) GluA1, Synapsin 1, GluA1, and phospho (p)‐Ser‐845 GluA1 (Thermo Fisher Scientific) at a 1:1000 dilution at 4°C overnight, and incubated with peroxidase‐conjugated secondary antibody (1:1000 dilution, at room temperature, 1 h) and developed with a ECL system (GE Healthcare, Little Chalfont, Buckinghamshire, United Kingdom). The relative intensity was calculated by correcting for β‐actin with NIH‐Image J1.51p 22.

### 2.9 Immunofluorescence

The CA3 region of rat hippocampus was embedded with Optimal Cutting Temperature™ medium (Richard‐Allan Scientific, Kalamazoo, MI) and snap‐frozen in liquid nitrogen. Five‐micrometer sections were cut by a Microm HM525 cryostat, which were fixed with 4% paraformaldehyde and blocked with 4% goat serum in phosphate‐buffered saline. Primary antibody against BDNF (1:200; Abcam, Cambridge, MA) was incubated at 4°C overnight, and Texas Red‐conjugated goat antimouse secondary antibody (1:500; Becton Dickinson, Franklin Lakes, NJ) were incubated for 2 h. The cell nucleus was stained with 4′,6‐diamidino‐2‐phenylindole (DAPI). Photos were taken with a Nikon 80*i* fluorescence microscope (Nikon, Tokyo, Japan).

### 2.10 Statistical analysis

One‐way ANOVA followed Dunn's multiple comparisons test, and Two‐way ANOVA followed Tukey's multiple comparisons test. The significance level was set at a *p*‐value < .05. All statistical analyses were performed using GraphPad Prism.

## 3 RESULTS

### 3.1 Enriched environment exposure ameliorates postsurgery sleep deprivation‐induced cognitive impairment

**FIGURE 1:**
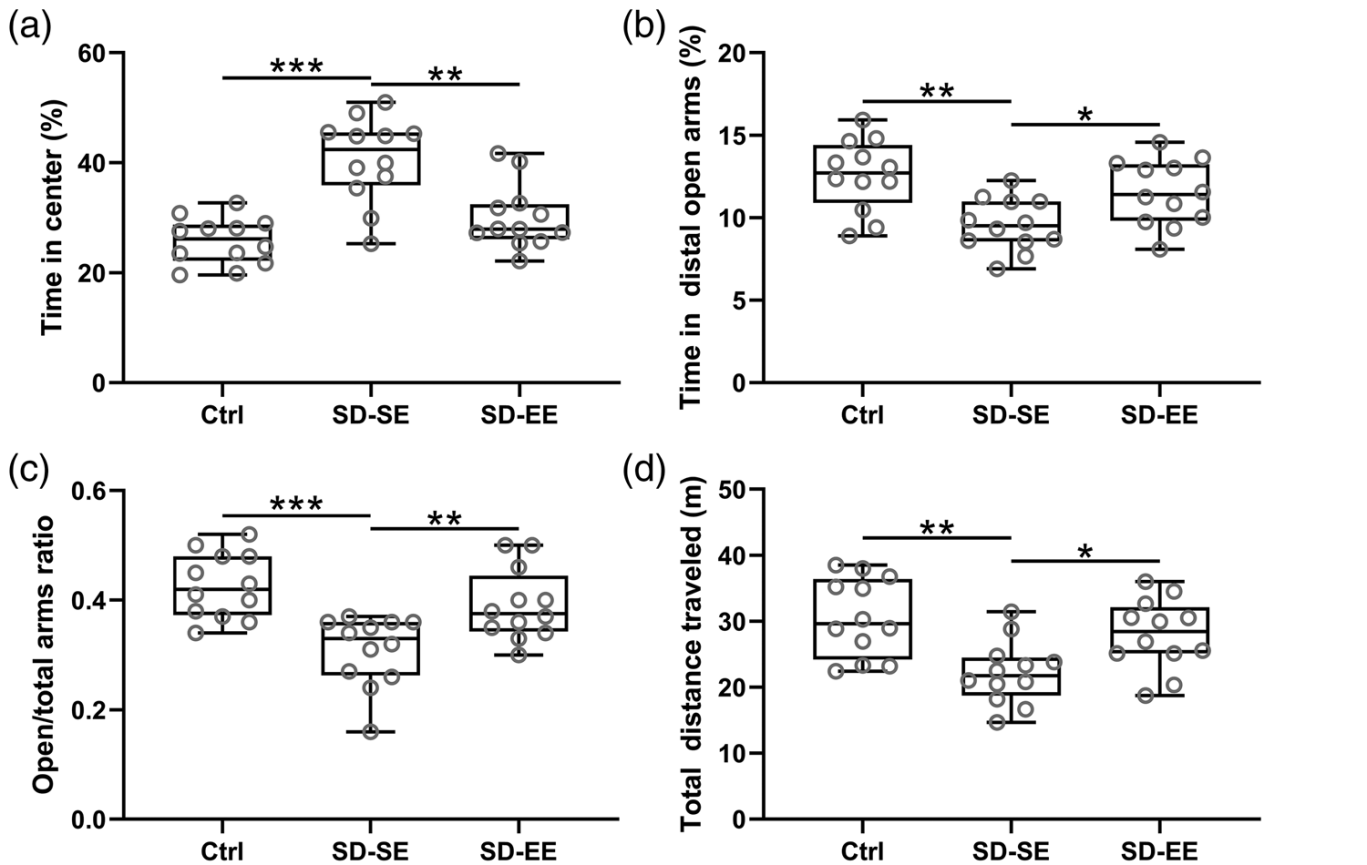
Exposure to enriched environment ameliorated postsurgery sleep deprivation‐induced cognitive impairment of rats in an elevated plus maze. Enriched environment (EE) exposure decreased the time spent in the center (a), improved the time spent in the distal open arms (b), open/total arms ratio (c), as well as total distance traveled (d) compared with standard environment (SE) exposure. *n* = 12 rats for each group. Data were shown with box plot. *p < .05, **p < .01, ***p < .001. One‐way ANOVA followed Dunn's multiple comparisons test.

EPM, NOR, OLM, and Morris Water Maze tests were applied to evaluate the cognitive impairment induced by SD after postsurgery. As expected, increased time spent in the center (Figure 1a), diminished time spent in the distal open arms (Figure 1b), decreased open/total arms ratio (Figure 1c), and reduced total distance traveled (Figure 1d) were observed in SD‐SE rats when compared with Ctrl rats, which indicated that postsurgery SD could induce cognitive impairment. On the other hand, damaged cognitive impairment induced by postsurgery SD could be reversed by EE exposure.

**FIGURE 2:**
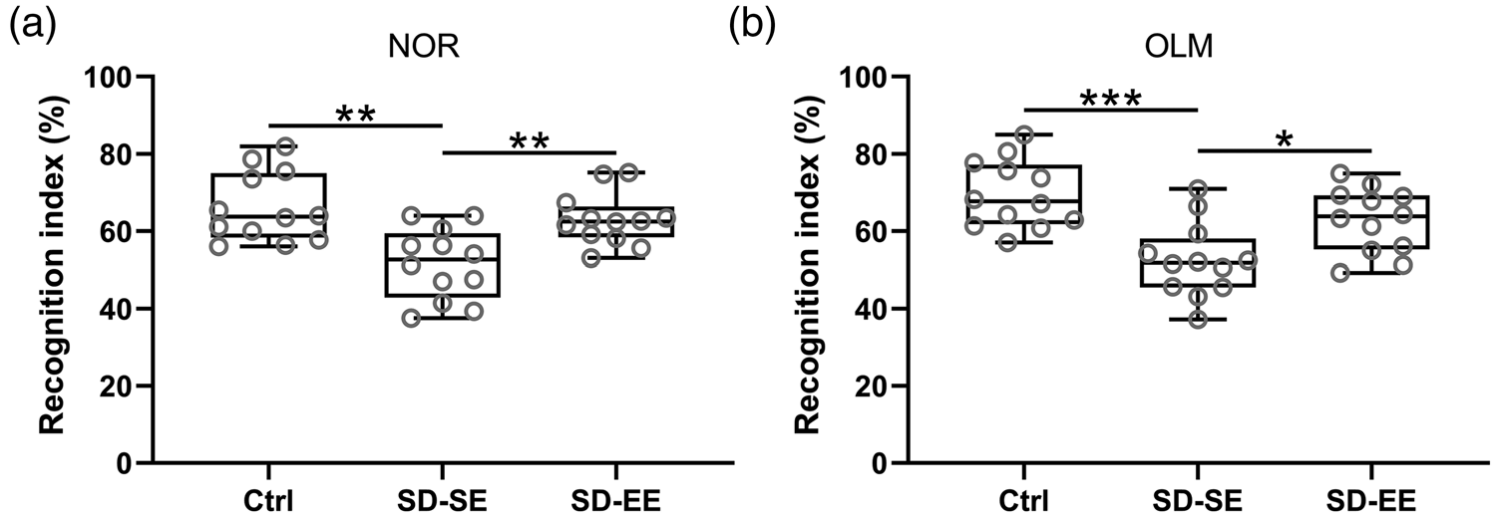
Exposure to enriched environment ameliorated postsurgery sleep deprivation‐induced cognitive impairment of rats in novel object recognition assay and object location memory task. Novel object recognition (NOR, A) or object location memory (OLM, B) were carried out 24 h after EE or SE exposure to objects. The recognition index was calculated as the proportion of time with the target or novel object out of the total time. *n* = 12 rats for each group. Data were shown with box plot. *p < .05, **p < .01, ***p < .001. One‐way ANOVA followed Dunn's multiple comparisons test.

**FIGURE 3:**
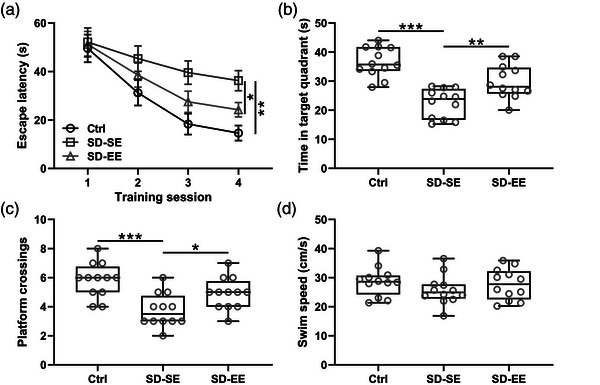
Exposure to enriched environment ameliorated postsurgery sleep deprivation‐induced cognitive impairment of rats in Morris Water Maze test. In four training sessions, escape latencies (a) and swim speed (d) were recorded. In the probe trial, the number of platform crossings (c) and time in the target quadrant in 60 s (B) were recorded. *n* = 12 rats for each group. Data were shown with box plot. *p < .05, **p < .01, ***p < .001. One‐way ANOVA followed Dunn's multiple comparisons test and Two‐way ANOVA followed Tukey's multiple comparisons test.

Decreased recognition index in the NOR test (Figure 2a) and reduced recognition index in the OLM test (Figure 2b) were observed in SD‐SE rats, and SD‐EE rats demonstrated the opposite trend. In the Morris Water Maze test, increased escape latencies (Figure 3a), decreased time in the target quadrant in 60 s (Figure 3b), and decreased numbers of platform site crossings (Figure 3c) were observed in SD‐SE rats when compared with Ctrl rats, while EE exposure could reverse such changes. It was worth noting that there was no significant difference in average swim speed among Ctrl rats, SD‐SE rats, and SD‐EE rats (Figure 3d). Our analysis testified that EE exposure ameliorated postsurgery SD‐induced cognitive impairment.

### 3.2 Enriched environment exposure ameliorates postsurgery sleep deprivation‐induced neuron loss

**FIGURE 4:**
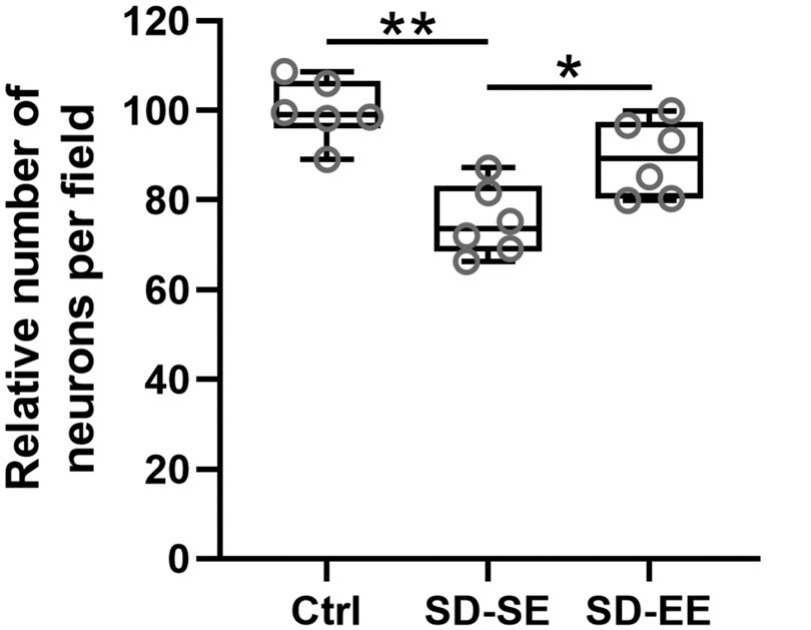
Exposure to enriched environment ameliorated postsurgery sleep deprivation‐induced neuron loss in rats. The relative number of neurons in the experimental groups. Ten fields in one rat, and six rats in each group were averaged. Scale bar = 50 μm. Data were shown with box plot. *p < .05, **p < .01. One‐way ANOVA followed Dunn's multiple comparisons test.

The CA3 region of the hippocampus was important for the rapid encoding of memory. In this study, the representing cresyl violet staining results of the CA3 region were demonstrated to indicate the potential neuron loss induced by postsurgery sleep deprivation. Decreased number of neurons was observed in SD‐SE rats when compared with Ctrl rats, while SD‐EE rats showed an upregulated number of neurons when compared with SD‐SE rats (Figure 4). These results demonstrated that postsurgery SD‐induced neuron loss could be alleviated by EE exposure.

### 3.3 Enriched environment exposure induces BDNF expression in the hippocampus of postsurgery sleep deprivation rats

**FIGURE 5:**
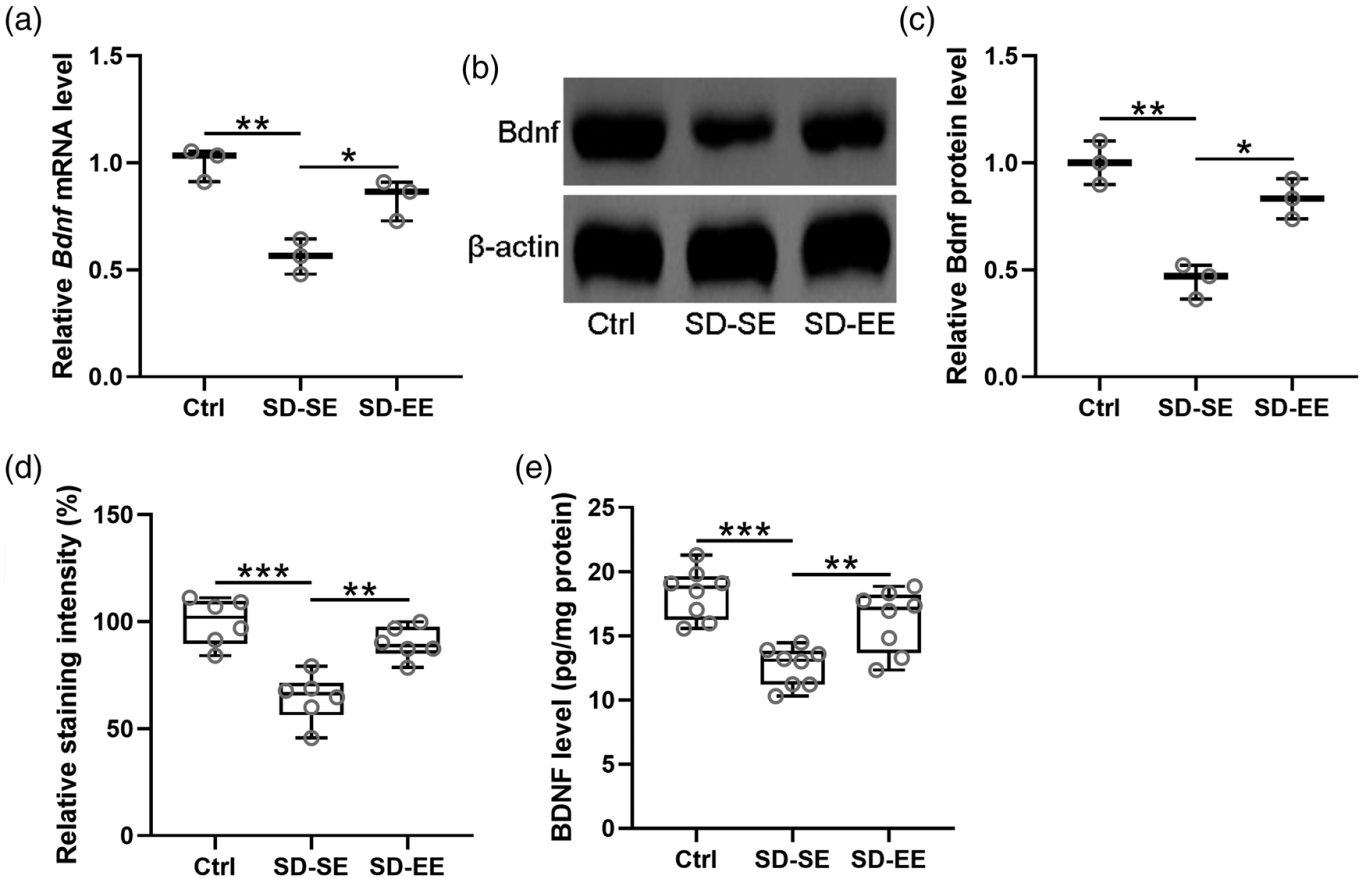
Exposure to enriched environment improved BDNF expression in the hippocampus of postsurgery sleep deprivation rats. The relative mRNA levels of Bdnf in the hippocampus were tested by RT‐qPCR (a). The relative protein levels of Bdnf in the hippocampus were assayed by Western blotting (b), and the expressions were normalized to β‐actin (c). *n* = 3 repeats for each group (8 hippocampus homogenates were mixed for each group). (d) Relative staining intensity of BDNF in CA3 region of rat hippocampus. Six rats in each group were used for staining. (e) The protein level of BDNF in the hippocampus was measured by ELISA. Six rats in each group were used. Data were shown with box plot. *p < .05, **p < .01, ***p < .001. One‐way ANOVA followed Dunn's multiple comparisons test.

The relative expression of BDNF in postsurgery SD was investigated. Decreased mRNA level (Figure 5a) and protein level (Figure 5b and c) of BDNF were observed in SD‐SE rats when compared with Ctrl rats, while SD‐EE rats demonstrated significantly increased BDNF expression when compared with SD‐SE rats. On the other hand, results demonstrated increased BDNF expression in SD‐EE rats compared with SD‐SE rats (Figure 5d). As expected, the protein level of BDNF in the hippocampus decreased in SD‐SE rats compared with Ctrl rats, while SD‐EE rats demonstrated significantly upregulated BDNF secretion compared with SD‐SE rats (Figure 5e). These results confirmed that EE exposure induced upregulated BDNF expression in the hippocampus of postsurgery SD rats, which might contribute to the alleviation of neuron loss.

### 3.4 Enriched environment exposure improves synaptic GluA1 subunit expression in the hippocampus of postsurgery sleep deprivation rats

**FIGURE 6:**
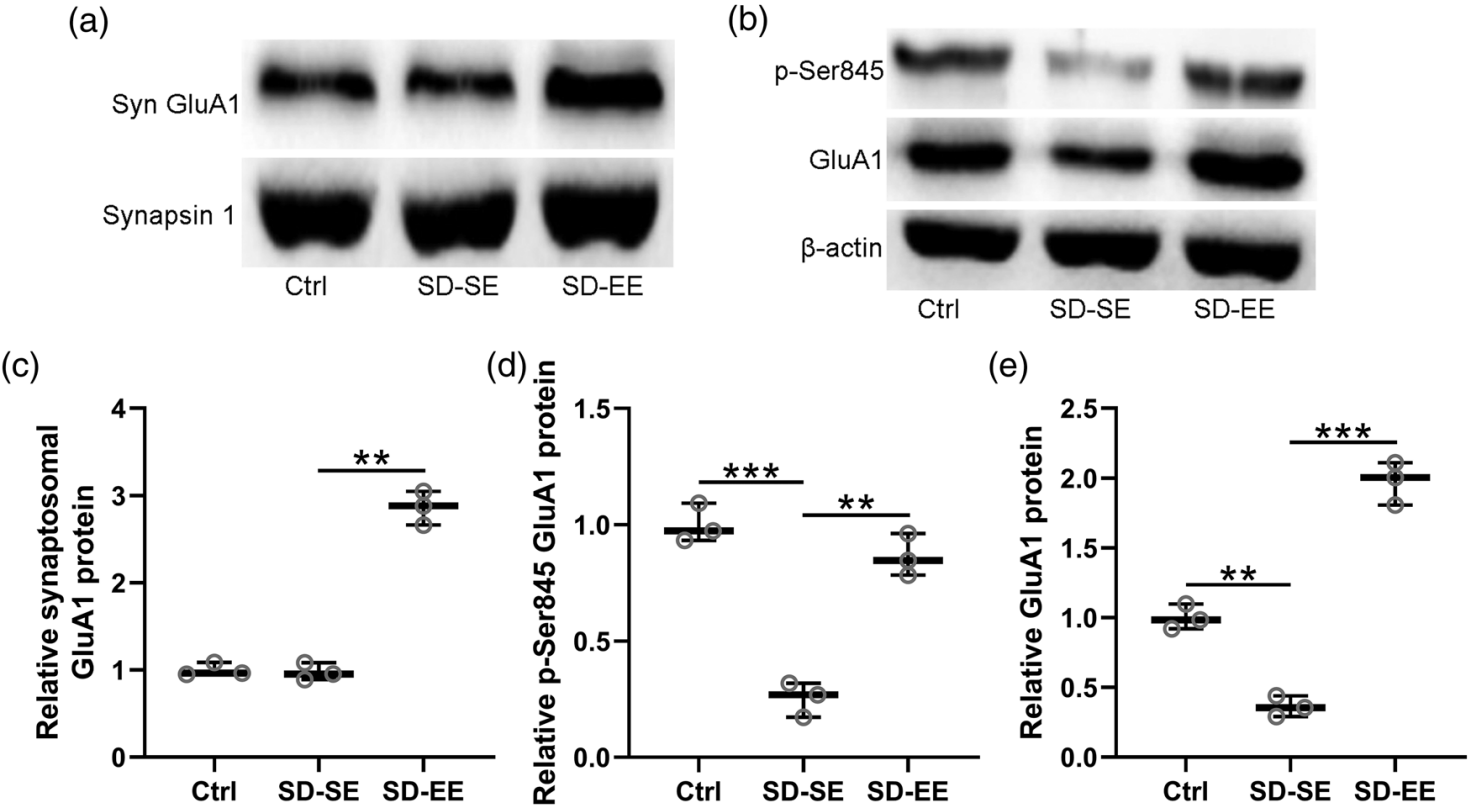
Exposure to enriched environment improved the level of synaptic GluA1 subunits in hippocampus of postsurgery sleep deprivation rats. (a) Synaptosomal (Syn) GluA1 proteins in the hippocampus were assayed with Western blotting. The relative expressions were normalized to Synapsin 1 (c). Phosphorylated GluA1 at Ser‐845 and total GluA1 proteins in the hippocampus were measured by Western blotting (b). The relative expressions were normalized to β‐actin (d and e). *n* = 3 repeats for each group (8 hippocampus homogenates were mixed for each group). Data were shown with box plot. **p < .01, ***p < .001. One‐way ANOVA followed Dunn's multiple comparisons test.

Syn GluA1 subunits (Figure 6a) and p‐GluA1 (Figure 6b) in the hippocampus of postsurgery SD rats were measured by Western blot. No significant Syn GluA1 expression was observed in Ctrl rats and SD‐SE rats, and SD‐EE rats demonstrated significantly upregulated expression (Figure 6c). As to p‐GluA1 at Ser‐845 (Figure 6d) and total GluA1 proteins (Figure 6e), SD‐SE rats demonstrated decreased expression compared with Ctrl rats, and SD‐EE rats demonstrated upregulated expression when compared with SD‐SE rats. These data indicated that EE exposure could induce synaptic GluA1 subunit expression in the hippocampus of postsurgery SD rats.

## 4 DISCUSSION

In this study, hernia repair surgery without skin/muscle retraction accompanied by sleep deprivation is utilized to mimic the clinical scenario of postsurgery SD. At the same time, EE or SE exposure is utilized further to investigate the role of environment on postsurgery SD. As indicated by EPM, NOR, OLM, and Morris Water Maze tests, EE exposure ameliorates postsurgery SD‐induced cognitive impairments and neuron loss with upregulated BDNF and p‐GluA1(Ser845) expression. This study further promotes the clinical utilization of EE to alleviate postsurgery SD.

EE stimulation can change the brain's structure and function across an animal's lifespan (Kempermann, 2019). The function gain may be attributed to the development and plasticity of the brain (Alipio et al., 2022; Baroncelli et al., 2010; Cintoli et al., 2018). Mechanically, EE exposure increases the myelination of subcortical pathways, promotes capillary perfusion, and dramatically boosts the interaction between astrocytes and synapses (Loe & Feldman, 2009). The hippocampus plays a crucial role in declarative memory and spatial navigation, which is also heavily involved in brain disorders such as epilepsy and Alzheimer's disease (Chung et al., 2021). Our study indicates that the loss of neurons in the hippocampus might contribute to the development of postsurgery SD‐induced cognitive impairments, which could be alleviated by EE exposure.

Acute BDNF treatment promotes the interactions between GluA1 and GluA2 with their scaffold proteins at synapses, which can prolong the long‐term maintenance of AMPAr subunits and associated scaffolding proteins (Jourdi & Kabbaj, 2013). On the other hand, BDNF activates the mammalian target of rapamycin (mTOR) to regulate GluA1 expression required for memory formation (Slipczuk et al., 2009), and BDNF can induce heterogeneous nuclear ribonucleoprotein (hnRNP) A2/B1‐mediated local translation of GluA1 (Jung et al., 2020). Although the detailed mechanism needs further analysis, combined with previous reports, our study demonstrates that BDNF could promote the maintenance, mTOR‐regulated GluA1 expression, and hnRNP A2/B1‐mediated local translation of GluA1.

Phosphorylate regulation of GluA1 (Ser831 or Ser845) may play a critical role in bidirectional synaptic plasticity. Either S845 or S831 alone may support long‐term potentiation and depression in the hippocampus, and promote enhanced emotional learning and activity‐dependent synaptic plasticity in the cortex (Hu et al., 2007; Lee et al., 2010; Vargas‐Caballero et al., 2022). It is worth noting that increased GluA1 expression and phosphorylation are common phenomena that happen during schizophrenia, depression, Alzheimer's disease, and chronic drug addiction (Sathler et al., 2021; Zhang & Abdullah, 2013). Although synaptic GluA1(Ser831) is not detected, our study demonstrates that GluA1 Ser845 phosphorylation could be induced by EE, which might lead to increased synaptic plasticity or decreased neuron loss.

Evidence has steadily been building for the function of cerebellum in cognition, emotional processing, and social behavior (Kwon, 2022). Cerebellum demonstrates sleep stage–dependent activity, and the malfunctions can result in sleep–wake cycle alteration, leading to sleep disorders. During the awake state and sleep state, cerebral cortex and cerebellum strongly interact to promote memory consolidation (Canto et al., 2017). It is now realized that cerebellum is of potential importance to a range of neuropsychiatric disorders, such as autism and schizophrenia (Low et al., 2021). The potential role of cerebellum in postsurgery SD‐induced cognitive impairments might be an exciting field to decipher.

Some limitations should be indicated here. Our investigation demonstrates that EE might promote BDNF/GluA1 interaction‐mediated cognitive improvement, while the conclusion needs further testification analysis with pathway inhibition assay. Only one model of cognitive impairment, open inguinal hernia repair surgery without skin/muscle retraction, is utilized in this investigation. The universal role of EE on postsurgery SD needs further detailed analysis. On the other hand, the role of EE on normal healthy animals is demonstrated in previous investigations (Córneo et al., 2022; Tooley et al., 2021), which should also be testified in our facility.

## 5 CONCLUSION

Enriched environment ameliorates postsurgery sleep deprivation‐induced cognitive impairments through BDNF/GluA1 pathway.

## CONFLICT OF INTEREST STATEMENT

The authors declared that they had no conflict of interest.

### PEER REVIEW

The peer review history for this article is available at https://publons.com/publon/10.1002/brb3.2992.

## Data Availability

Data will be made available on request.
